# Lymphocyte immunophenotype in dogs with immune-mediated hematologic disease

**DOI:** 10.1371/journal.pone.0326341

**Published:** 2025-06-17

**Authors:** Shauna L. Blois, Benoît Y. Cuq, Dorothee Bienzle

**Affiliations:** 1 Department of Clinical Studies, Ontario Veterinary College, University of Guelph, Guelph, Ontario, Canada; 2 Department of Pathobiology, Ontario Veterinary College, University of Guelph, Guelph, Ontario, Canada; University of Split Faculty of Medicine: Sveuciliste u Splitu Medicinski fakultet, CROATIA

## Abstract

Immune-mediated hematologic diseases (IMHD) including immune-mediated hemolytic anemia (IMHA) and immune thrombocytopenia (ITP) can cause severe disease in dogs. The underlying immune system abnormalities associated with these conditions is not known. The hypotheses of this study were that dogs with IMHD would have increased frequencies of CD4+ and CD8 + lymphocytes, decreased frequencies and numbers of T regulatory cells, and increased frequencies of interleukin (IL)-17 + lymphocytes. Fifteen dogs with newly diagnosed IMHA or ITP and 15 healthy control dogs were recruited for this prospective study. Flow cytometry was used to enumerate CD4 + lymphocytes, CD8 + lymphocytes, T regulatory (CD4 + CD25 + Foxp3+) cells, and lymphocytes secreting IL-17 in dogs with IMHD at diagnosis, then 2 and 4 days after starting immunosuppressive treatment. Median proportion of CD4+ (Day 0: 3.4%, Day 2: 3.3%) and CD8+ (Day 0: 1%, Day 2: 0.6%) cells was lower in dogs with IMHD compared to control dogs (CD4 + 22.8%, CD8 + : 13.6%; P < 0.0001 for each). Additionally, T regulatory cells were reduced in IMHD dogs at Day 0 (0.2% versus 0.6% of total lymphocytes, P = 0.0025). Dogs with IMHD had a higher proportion of lymphocytes positive for IL-17 at Day 2 (1.3%) compared to control dogs (0.4%, P = 0.0024). Dogs with IMHD have immune system alterations at diagnosis and during early treatment characterized by a deficiency in T regulatory cells and an increase in IL-17 + lymphocyte. These changes might contribute to the pathogenesis of IMHA and ITP.

## Introduction

Immune-mediated hemolytic anemia (IMHA) and immune thrombocytopenia (ITP) are common immune-mediated hematologic diseases (IMHD) in dogs. Both IMHA and ITP can cause severe disease necessitating hospitalization in the intensive care unit, transfusion of blood products, and immunosuppressive treatment [1–6]. These conditions in dogs share pathogenic features with warm autoimmune hemolytic anemia (AIHA) and ITP in people [7,8]. Although extensively studied, the pathogenesis of AIHA and ITP in people, as well as canine IMHA and ITP, are poorly understood.

The pathogenesis of IMHD is likely complex and multifactorial. Both AIHA and ITP are thought to arise at least in part from excessive autoantibody production against erythrocytes or platelets, respectively [9,10]. CD4 + T helper cells contribute to the autoantibody production from B cells. Clonal expansion of cytotoxic CD8 + cells is found in people with AIHA and ITP. While the role of such CD8 + cells in the pathophysiology of these conditions is not elucidated, they might contribute to an antibody-independent method of platelet destruction in ITP [7,11–13]. People with IMHD also have defective T regulatory cell responses [13–16].

Interleukin (IL)-17 is a pro-inflammatory cytokine involved in recruiting and activating neutrophils, monocytes, epithelial cells, and endothelial cells [17]. IL-17 is produced predominantly by a subset of CD4 + T helper cells called Th17 cells [18–21]. IL-17-producing cells and excess IL-17 concentrations have key roles in the pathogenesis of autoimmune diseases in people [14,19,22–24]. People with AIHA and ITP have increased numbers of Th17 cells in circulation along with increased plasma IL-17 concentration, compared with healthy individuals [15,23,25,26].

There is a paucity of information regarding the immune system changes involved in the pathogenesis of canine immune mediated diseases overall, and in particular IMHD. One previous study of dogs with IMHA did not show changes in lymphocyte subsets compared to healthy dogs or dogs with other illnesses [27]. In a separate study, dogs with IMHA that did not survive the acute phase of disease had persistently higher serum IL-17 concentration compared to surviving dogs [28]. However, comprehensive investigation into the immune cell abnormalities, and the potential role of IL-17, in dogs with IMHD is lacking.

The objective of this study was to describe the prevalence of different T cell populations, including CD4+ and CD8 + lymphocytes, T regulatory cells, and IL-17 positive lymphocytes in dogs with IMHA or ITP at diagnosis and up to 4 days following initiation of treatment. Study hypotheses were that compared to healthy individuals, dogs with newly diagnosed IMHA and ITP would have: (1) increased proportions and numbers of CD4+ and CD8 + lymphocytes; (2) decreased proportions and numbers of T regulatory (CD4 + CD25 + Foxp3+) cells; (3) increased proportions and numbers of IL-17 + lymphocytes, and (4) that these changes would persist for up to 4 days after the start of treatment.

## Materials and methods

### Ethics statement

Blood samples were obtained from client-owned dogs for this study, after informed written client consent was obtained. All protocols in this study were in accordance with institutional animal use guidelines and approved by the Animal Care Committee of the University of Guelph (Protocol Number: 3789). All dogs in the study had access to standard of care treatment for IMHD, in accordance with published guidelines, and care was dictated by the primary clinician and dog owner [3]. No changes to treatment were instituted by participation in the present study. The study protocol involved venipuncture and collection of blood at up to 3 separate time points from all enrolled dogs. No analgesia was provided for study purposes as this is not standard practice for venipuncture in dogs. Humane euthanasia was not performed for study purposes. However, humane euthanasia via intravenous injection of pentobarbital was opted by some clients based on severity of disease, or due to lack of response to standard of care treatment, and this occurred separate from the study protocol.

### Study population

This study included 15 client-owned dogs diagnosed with IMHD presenting to the Ontario Veterinary College Health Sciences Centre and 15 client-owned healthy dogs. Inclusion criteria for dogs with IMHD were as follows: no immunosuppressive or transfusion therapy prior to enrolment, written consent obtained from client, dog amenable to venipuncture for study purposes, and diagnosis of non-associative IMHA or ITP (defined in following statements). Dogs were classified as having a diagnosis consistent with IMHA if the following criteria were met: (a) presence of anemia (hematocrit <0.39 L/L), (b) at least one sign of immune-mediated red cell lysis (spherocytosis, persistent autoagglutination after saline washing, positive direct antiglobulin test), (c) at least one sign of hemolysis (hemoglobinemia, hemoglobinuria, presence of red cell ghost cells on a fresh smear, hyperbilirubinemia or bilirubinuria in absence of hepatobiliary disease or sepsis) [29]. Immune thrombocytopenia was diagnosed based on a platelet count <50,000/μL confirmed on blood smear review, hemorrhage and disseminated intravascular coagulation ruled out with appropriate examination, imaging, and coagulation testing [30]. Dogs were investigated for underlying triggers of IMHA or ITP based via a complete blood cell count (CBC) including blood smear review by a clinical pathologist, serum biochemical profile, urinalysis, thoracic radiographs, abdominal ultrasound, and infectious disease testing relevant to individual travel history using a Snap 4Dx Plus (IDEXX Laboratories Canada, Markham, Ontario) [29,30]. This information was used to classify the diagnosis as “probable,” “supportive of,” or “diagnostic for” IMHA or ITP [29,30]. Exclusion criteria were administration of any immunosuppressive therapy prior to enrolment, including glucocorticoids, cyclosporine, mycophenolate, oclacitinib, lokivetmab, or similar medications, or administration of blood transfusion prior to enrolment. Dogs were also excluded if they were suspected to have associative IMHA or ITP based on the above diagnostic test results, such as the presence of vector-borne disease, neoplasia, or other concurrent inflammatory diseases.

Healthy control dogs were recruited from staff- and student-owned dogs at the study institution. Inclusion criteria were age between 1 and 8 years, unremarkable health history for the previous 1 year, up to date on recommended core vaccination and parasite prevention for the region, normal physical examination, and normal minimum database (CBC, serum biochemical profile, free catch urinalysis). Exclusion criteria were administration of any medications outside of recommended parasite prophylaxis, vaccination administration within the past 2 months, and any previous suspicion or diagnosis of immune-mediated or hypersensitivity disorder including atopic skin diseases, or other major illness.

An estimated sample size was calculated using data from a previous study measuring frequencies of CD4 + IL-17 + cells in people with AIHA [14]. Based on estimates of 0.25% (+/-0.2) and 3.25% (+/-1) of CD4 + IL-17 + cells in controls and cases, respectively, and assuming normal data distribution with a logit transformation followed by a t-test with (alpha 0.05, power 90% or more), at least 8 dogs in each group was recommended. Due to the unknown differences in canine populations, the consulting statistician recommended increasing the study number to 10–15 dogs to help identify smaller population differences or differences within a more heterogeneous population.

Blood was sampled via direct jugular or saphenous venipuncture and collected into tubes containing EDTA for CBC and flow cytometry, and a serum separator tube for biochemical analysis. EDTA blood was refrigerated for up to 72 hours prior to flow cytometry analysis. In the validation process for the flow cytometry protocol, a small number of stored samples were analyzed daily for 3 days. Proportions and numbers of lymphocytes were similar across the 3 days. Based on those trials and the previous experience with canine samples in our institution’s flow cytometry lab, a sample storage time of up to 72 hours was considered appropriate. When available, blood samples from dogs with IMHD on Day 2 and 4 after initiation of treatment were analyzed.

### Cell preparation and flow cytometry

In order to focus the investigation on leukocytes, red blood cells were lysed using freshly diluted red blood cell (RBC) lysis buffer (RBC Lysis Buffer 10X, eBioScience, Thermo Fisher Scientific), as per manufacturer’s recommendations. EDTA-anticoagulated whole blood (100 μL) was added to 1.5 mL RBC lysis buffer, vortexed, then incubated at room temperature for 10 minutes. The sample was washed twice by adding 1 mL of flow buffer, vortexing, centrifuging at 1200 rpm for 5 minutes, then decanting. Concanavalin A was added to each tube for a final concentration of 2.5 μg/mL. Concurrently, protein transport inhibitor (eBioscience Protein Transport Inhibitor Cocktail 500X) was added to each tube for a final concentration of 2 μL/mL. Tubes were incubated in the dark at 4^o^C for 2 hours.

Four tubes per individual were analyzed in parallel. Antibody clones and fluorochrome conjugates are listed in Table 1. In tube 1 (unstained), no antibody was added. Tube 2 received 100 μL of viability stain (Zombie NIR fixable viability kit, BioLegend, San Diego, CA). Surface antibodies were added in the following manner: 1 μL of anti-CD4 and 3 μL of anti-CD8 to tube 3, and 1 μL of anti-CD4 and 3 μL of anti-CD25 to tube 4 (all antibodies from eBioscience); tubes were incubated in the dark at room temperature for 10 minutes prior to washing. One mL of cell fixative (True-Nuclear 1X Fix Concentrate, BioLegend) was then added to tubes 2–4. All tubes were vortexed and incubated at 4 °C for 12-18h. Then 2 mL of cell permeabilization buffer (True-Nuclear 1X Perm Buffer, BioLegend) was added to each tube to facilitate intracellular staining for IL-17 and Foxp3. The tubes were centrifuged at 1200 rpm for 5 min and the supernatant decanted. The pellet was resuspended in 100 μL of cell permeabilization buffer, and 3 μL of anti-Foxp3 and 5 μL of anti-IL-17A (both from eBioscience) were added to tubes 3 and 4. All tubes were incubated in the dark at room temperature for 30 minutes. Two mL of cell permeabilization buffer was added to the tubes, then the tubes were centrifuged at 1200 rpm for 5 minutes and the supernatant decanted. Two mL of cell staining buffer was added to each tube, prior to centrifugation, decanting, and resuspending the pellet in 300 μL of flow buffer. Tubes were stored in the dark at 4 °C for up to 1 hour until analysis on a flow cytometer. All antibody concentrations were based on previous trials titrating the optimal volume of antibody for separating positive and negative cell signals. The antibody clones used for CD4, CD8, CD25 are anti-canine antibodies and have been used in previous trials in our facility’s laboratory as well as in others’ published research [31–34]. The Foxp3 antibody is not an anti-canine antibody but has been shown to have reactivity with canine lymphocytes [35]. The IL-17 antibody clone is not specific for dog IL-17. This clone was selected because it was previously reported to detect canine IL-17 by flow cytometry with concurrent increases of IL-17 detected via ELISA [21].

**Table 1:** Antibody clones used in flow cytometry analysis.

Target antigen	Clone, host, isotype	Fluorochrome
CD4	YKIX302.9, rat, IgG2a, kappa	FITC
CD8	YCATE55.9, rat, IgG1, kappa	APC
CD25	P4A10, mouse, IgG1, kappa	eFlour 660
Foxp3	FJK-16s, rat, IgG2a	PE
IL-17A	eBio17B7, rat, IgG2a, kappa	eFluor 450

CD, cluster of differentiation; Foxp3, forkhead protein box 3; IL, interleukin; IgG, immunoglobulin G; FITC, Fluorescein isothiocyanate; APC, allophycocyanine; PE, phycoerythrin.

Immediately prior to analysis, samples were briefly vortexed, then analyzed using a multi-laser flow cytometer (FACSCanto II, BD Biosciences). At least 10,000 events were acquired per tube. Gates were set to include all leukocytes based on forward and side scatter properties. Unstained negative control samples were used for each stained sample analyzed. Viability stain results were used to modify the gate to include only viable cells. Data was analyzed using commercial software (FlowJo v10.8 Software, BD Biosciences).

**Fig 1:**
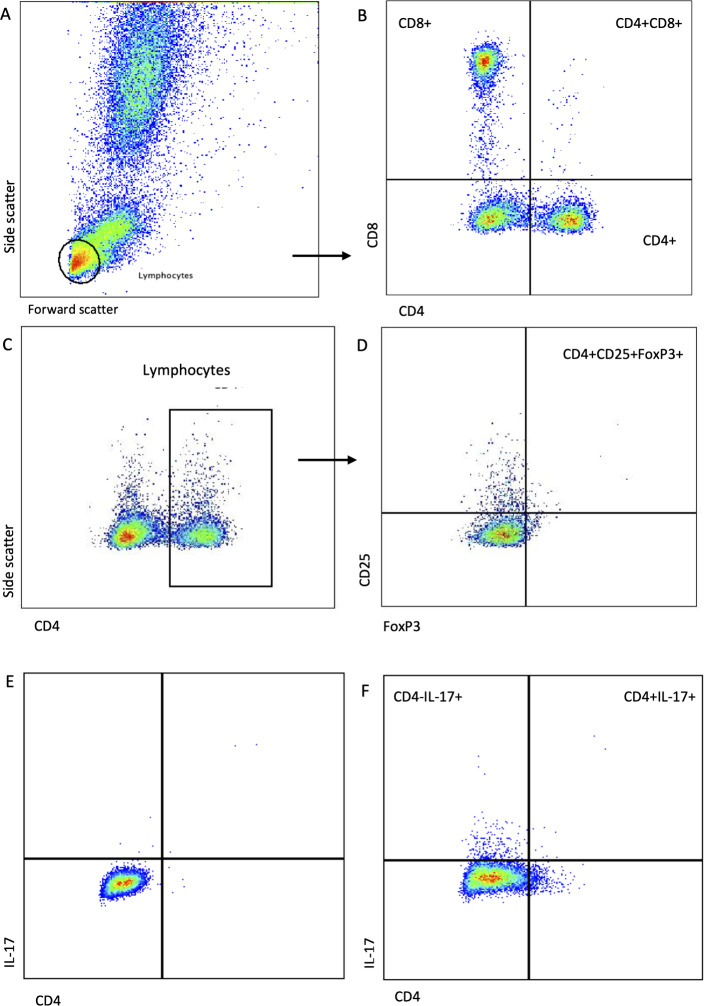
Characterization of lymphocyte subsets using flow cytometry. Lymphocytes were identified based on forward and side scatter characteristics (A) and then gated for further analysis. Gating for positive events was based on unstained samples for each dog, where the negative quadrant was set to include >99.5% of cells. The gating strategy was used to identify CD4+ and CD8 + cells (B). CD4 + cells were gated from the lymphocyte population (C) and then further evaluated for CD25+ and Foxp3 + positivity to show T regulatory cells (D). An unstained control is shown in (E) to compare the IL-17 positive lymphocytes in (F). The total number of IL-17 positive lymphocytes was derived from the CD4- and CD4 + populations that were considered positive for IL-17, depicted in the two upper quadrants of this image.

To identify CD4 + , CD8 + , and IL-17 + cells, the lymphocyte population was identified in dot plots based on forward and side light scatter properties. The lymphocytes were then gated for further analysis. Unstained control samples were used to set CD4 and CD8 gates such that >99.5% of events were contained within the negative quadrant. The CD4 + cells were further examined for CD25 and Foxp3 (Fig 1). Total lymphocyte IL-17 positivity was examined in the CD4+ plus CD4- cells (Fig 1). The percent positive for each population of cells was determined by setting the quadrant on the negative control sample to include >99.5%. Absolute cell counts were calculated for all dogs by multiplying the proportion of the subset within the lymphocyte gate by the total lymphocyte count from the CBC. Lymphocyte counts were inconsistently available at Day 4, therefore absolute counts were not calculated for these days. Gates with positive versus negative events were established during experimental design studies using fluorescence-minus-one (FMO) controls for each antibody combination tube.

### Statistical analysis

Descriptive statistics for the population data of dogs with IMHD and control dogs were calculated. Normality was assessed using a Shapiro Wilk test. Differences between the IMHD and control groups were assessed with t-tests.

To analyze CD4 + , CD8 + , T regulatory, and IL-17 positive lymphocyte results, the data was again assessed for normality. Both absolute counts and relative proportion of each lymphocyte subpopulation was assessed. Descriptive data including mean + /- SD or median and interquartile range (IQR) were calculated for both study groups at each time point, for parametric and non-parametric data, respectively. Next, each parameter was compared between IMHD and control dogs at Day 0 and Day 2 using parametric (one-way ANOVA) or non-parametric (Kruskal Wallis) tests, depending on data distribution.

Analysis was performed using statistical software (GraphPad, Version 10.2.2).

## Results

### Study population

Samples were acquired from 15 dogs with IMHD and 15 control dogs between January 2018 and August 2019. Study population details are found in Table 2. Of the dogs with IMHD, Day 0 samples were collected from 14 dogs (11 dogs with IMHA, 3 dogs with ITP), Day 2 samples were collected from 10 dogs (8 dogs with IMHA, 2 dogs with ITP); and Day 4 samples were collected from 5 dogs (4 dogs with IMHA, 1 dog with ITP). One control sample was removed from analysis of the CD4 + , CD8 + , and IL-17 + cells due to poor sample quality. Data was not available from one dog (IMHA Day 0) for CD4 + CD25 + Foxp3 analysis. Statistical analysis was not performed on Day 4 samples due to the small number but results from Day 4 are included in the graphs.

**Table 2:** Details of dogs included in the IMHD and control populations of the study.

Breed	Sex	Weight	Age	Condition
Standard schnauzer	FS	17	5	IMHA
Mixed breed	FS	4.3	8.5	IMHA
Mixed breed	FS	22.45	8.7	IMHA
Mixed Breed	MC	8.55	6.9	IMHA
Mixed breed	MC	20.75	8.9	IMHA
Min Schnauzer	FS	7.2	11	IMHA
Mixed-breed	FS	8	9.6	IMHA
Mixed breed	FS	21.3	6	IMHA
Mixed breed	MC	11.3	7.1	IMHA
Mixed breed	FS	26.2	12.1	IMHA
Mixed breed	MC	13	8	IMHA
Mixed breed	MC	5.5	2.6	IMHA
Pug	MC	13.2	6	ITP
Cocker Spaniel	MC	15.55	4	ITP
Mixed breed	MC	8.95	9	ITP
Beagle	MC	20	4.3	Control
Mixed-breed	FS	23	2.5	Control
Mixed-breed	MC	40.4	8.2	Control
Mixed-breed	MC	12	7.1	Control
Mixed-breed	MC	35	4.1	Control
Standard Poodle	MC	21.5	4.1	Control
Labrador Retriever	MC	35	4.3	Control
Labrador Retriever	MC	32.4	1.3	Control
Border Collie	FS	20	6.6	Control
Bernese Mountain Dog	FS	39.5	3	Control
Bernese Mountain Dog	MC	44.4	3	Control
Labrador Retriever	FS	33.2	6.3	Control
Shiloh Shepherd	MC	34.5	4.5	Control
Bulldog	FS	25	8.7	Control
Mixed-breed	FS	21.3	9.8	Control

FS, female spayed; IMHA, immune mediated hemolytic anemia; ITP, immune thrombocytopenia; MC, male castrated.

Dogs with IMHD were older (mean 7.6 + /-2.6 versus 5.2 + /- 2.5 years, p = 0.015) and smaller (mean 13.6 + /- 6.8 versus 29.1 + /- 9.4 kg, p < 0.0001) compared to control dogs. Of the 15 dogs with immune-mediated disease, 4 dogs with IMHA died or were euthanized within 2 days of presentation due to disease severity. Of the 12 dogs with IMHA, 10 met the “diagnostic for IMHA” and two met the “supportive for IMHA” criteria. Three dogs had ITP, 2 were considered “probable for ITP” and one was considered “diagnostic for ITP.” [29,30] All dogs were negative for infectious disease testing considered relevant to the geographic area. Of the dogs with IMHD, 12/15 were considered non-associative based on no underlying disease evident on thoracic radiographs and abdominal ultrasound (11) or post-mortem examination (1). Three dogs with IMHD had negative infectious disease testing and normal point-of-care thoracic and abdominal ultrasound, but comprehensive imaging (thoracic radiographs or abdominal ultrasound) was not performed. All dogs with immune-mediated disease were started on immunosuppressive corticosteroid therapy within 24 hours of hospital admission. Treatment decisions were made by the primary clinician and not standardized for the study. Individual dog details, including diagnostic test results and treatments, are included in S1 Table. All data was non-parametric in distribution and analyzed with non-parametric tests.

### Proportion of T cell subsets

Summarized data (median, interquartile range, and statistical analysis between subpopulations) of all evaluated lymphocyte populations is presented in Table 3.

**Table 3:** Frequency of CD4 + , CD8 + , and CD4 + CD25 + Foxp3 + cells is in proportion to the lymphocyte population.

		Frequency			Absolute number (uL)		
		Median	IQR	P-value	Median	IQR	P-value
CD4 + Lymphocytes	IMHD Day 0	3.4	0.9-10.6	**<0.0001**	49.2	21.7-136.6	**0.0002**
	IMHD Day 2	3.3	0.9-8	**<0.0001**	24.8	12.7-131.2	**0.0002**
	IMHD Day 4	5.3	2.5-12.4	---	134.6	83.1-263	---
	Control	22.8	18-32.2	---	487.0	289.3-630.8	---
CD8 + Lymphocytes	IMHD Day 0	1.0	0.6-2.4	**0.0003**	19.1	8.5-32.6	**0.0005**
	IMHD Day 2	0.6	0.3-1.7	**<0.0001**	6.7	0.5-20.7	**<0.0001**
	IMHD Day 4	0.4	0.2-2.4	---	10.6	4.8-67.7	---
	Control	13.6	5.8-27.3	---	284.1	124.9-496.4	---
CD4 + CD25 + Foxp3 + Lymphocytes	IMHD Day 0	0.2	0-0.3	**0.0038**	2.5	0.5-3.6	**0.006**
	IMHD Day 2	0.2	0.1-0.5	**0.0404**	3.3	1.2-6.6	0.1852
	IMHD Day 4	0.1	0.1-0.6	---	3.2	2.7-16.4	---
	Control	0.6	0.2-1	---	11.83	4.6-19.8	---
CD4 + IL-17 + Lymphocytes	IMHD Day 0	0.5	0.2-0.8	0.6742	5.6	2.3-11.4	**0.0135**
	IMHD Day 2	0.7	0.4-1.4	**0.0383**	12.0	3.3-18.6	**0.0042**
	IMHD Day 4	0.7	0.5-1.3	---	13.3	5-18.5	---
	Control	0.26	0.2-0.5	---	0.5935	0.4-3.1	---
CD8 + IL-17 + Lymphocytes	IMHD Day 0	0.4	0.2-0.6	**0.0023**	5.6	2.3-11.4	0.0559
	IMHD Day 2	0.5	0.2-0.9	**0.0003**	9.8	0.9-17.1	0.2501
	IMHD Day 4	0.5	0.4-0.7	---	13.3	5-18.5	---
	Control	0.035	0-0.1	---	0.7134	0.4-4.4	---

Frequency of CD4 + , or CD8 + , IL17 + lymphocytes refers to the percentage of the CD4 + , or CD8 + , lymphocyte population. All p values are in comparison to the control population. All data distribution is non-parametric.

**Fig 2:**
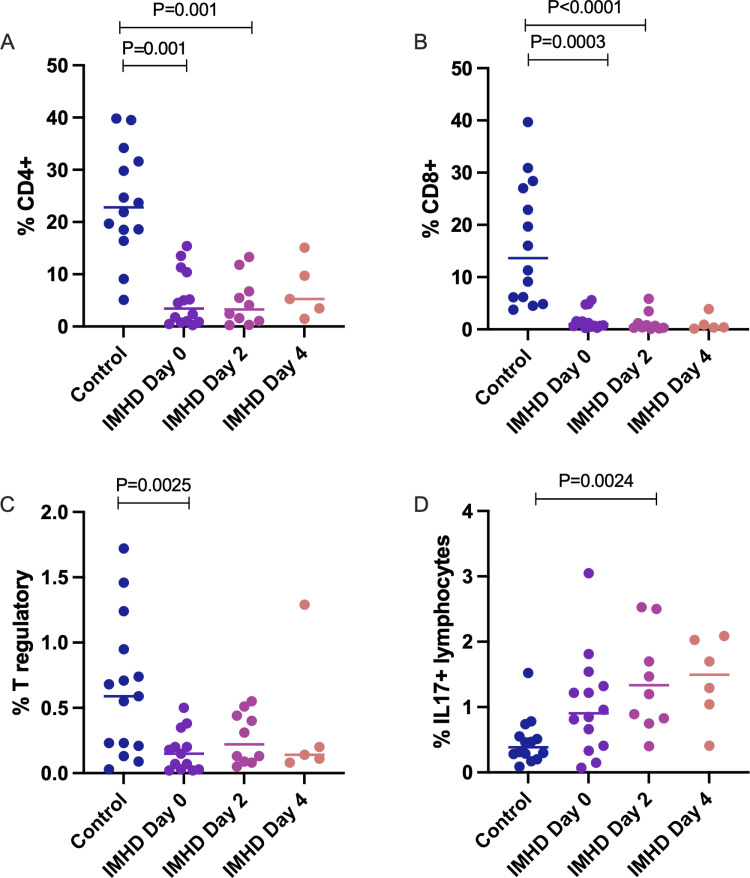
Proportion of lymphocyte subsets in the total lymphocyte population of health dogs and dogs with IMHD. Proportion of CD4+ (A), CD8+ (B), CD4 + CD25 + Foxp3+ (T regulatory cells; C), and IL-17+ (D) cells is as a percentage of the total lymphocyte population. All p values are in comparison to the control population. All data distribution is non-parametric. (CD, cluster of differentiation; Foxp3, forkhead box protein 3; IL, interleukin.).

The frequencies and absolute numbers of CD4+ and CD8 + cells were significantly higher in control compared to dogs with IMHD at Day 0 and Day 2 (Table 2, Figs 2a and 2b). Dogs with IMHD had significantly lower proportion (percent of total lymphocytes) and absolute number of CD4 + CD25 + Foxp3 + cells at Day 0 compared to control dogs (Table 2, Fig 2c). The total proportion of IL-17 + lymphocytes was greater in dogs with IMHD at Day 2 compared with control dogs (Fig 2d), while absolute numbers of IL-17 + lymphocytes were not different between groups (Table 2).

## Discussion

Immune mediated diseases are thought to arise from several changes in the immune response, including a loss of regulatory functions and excessive pro-inflammatory responses [27,36–38]. The present study identified novel aspects of the immunophenotype of dogs with IMHD at diagnosis and in the early phases of treatment. In particular, dogs with IMHD had lower CD4 + CD25 + Foxp3 + T regulatory cells than control dogs, and more lymphocytes positive for the pro-inflammatory cytokine IL-17 at one time point early in disease. Such findings suggest that a lack of T regulatory cells along with excessive pro-inflammatory IL-17 could play important roles in the pathophysiology of IMHD in dogs.

T regulatory cells have a vital function in promoting immune tolerance and homeostasis through their effects on immune cell activation and proliferation, as well as the effector functions of immune cells such as modulating cytokine expression [39,40]. The loss of such regulatory functions can lead to excessive immune-mediated responses and is commonly implicated in the pathogenesis of immune-mediated diseases. Studies of people with AIHA and ITP have identified decreased numbers and function of T regulatory cells compared to healthy people [15,41,42]. However, this is an inconsistent finding with some studies showing similar levels of T regulatory cells in healthy individuals and those with immune mediated disease [43–45]. The quantity of peripheral blood T regulatory cells does not necessarily reflect target organ effects, nor their actual function [41,43,44].

Dogs with IMHA had similar proportion of T regulatory cells compared with control dogs and dogs with non-immune inflammatory diseases in a previous study [27]. Differences between findings in that study and data presented here may be due in part to variable approaches to identify T regulatory cells, namely the identification of T regulatory cells as CD5 + CD4 + Foxp3 + cells in the previous study [27]. T regulatory cells are a subset of CD4 + cells and appear to be heterogeneous in both phenotype and function. While the definitions of T regulatory cell are variable, most include detection of the IL-2 receptor CD25, and the transcription factor Foxp3 [35,46,47]. In healthy dogs, CD4 + CD25^high^ cells are enriched for Foxp3 and show in vitro regulatory activity [35,47]. Thus, the present study defined T regulatory cells as those positive for CD4, CD25, and Foxp3 due to the data showing regulatory activity of these cells. This differs from previous research in dogs with IMHA where T regulatory cells were deduced to be CD4 and Foxp3 positive [27], potentially making the present study a more specific investigation of the role of T regulatory cells in IMHD. However, regulatory T cells likely have a variable phenotype and measuring one panel of cell markers might not fully represent the population of T regulatory cells [47,48]. Due to the relatively low number of CD4 + cells in dogs with IMHD here, it was difficult to reliably identify a CD4 + population that also had high CD25 positivity, as often the cell counts within these gates were low.

Interleukin-17 subtypes are pro-inflammatory cytokines, recruiting and activating various cells and inducing expression of other cytokines to increase inflammation. While often considered to be a product of a CD4 + subset (Th17 cells), IL-17 is produced by many different immune cells [49–53]. Overproduction of IL-17 has been linked to several immune-mediated diseases in people, including AIHA and ITP [14,23,28,53–55]. However, fewer studies have investigated the role of IL-17, or of the cells producing this cytokine in the pathophysiology of immune mediated diseases of dogs. Dogs with persistently higher serum levels of IL-17 were less likely to survive to hospital discharge, while high levels of IL-17 were detected in cerebrospinal fluid of dogs with steroid responsive meningitis arteritis [28,54]. In the present study, a higher proportion of lymphocytes producing IL-17 was observed in dogs with IMHA and ITP only at Day 2. Furthermore, the absolute count of IL-17 producing lymphocytes was not different between control dogs and those with IMHD in the present study. The absolute counts might be more relevant, as proportions are affected by other factors that may increase or decrease absolute lymphocyte concentration. While the increased proportion of IL-17 producing lymphocytes at one time point in the dogs with IMHD could suggest a contribution of this pro-inflammatory cytokine to disease pathogenesis, this finding requires further investigation in larger population as well as over longer time periods following initiation of treatment.

The relatively low proportion of CD4+ and CD8 + lymphocytes in dogs with IMHD in the present study was somewhat unexpected. Many components of the immune system are involved in the pathogenesis of immune-mediated disorders, and both CD4+ and CD8 + cells are considered to have key roles. CD4 + helper T cells have been shown to mediate the production of autoantibody from B cells in AIHA and ITP [56–58]. Depletion of CD4 + cells in a murine model of AIHA mitigated immune-mediated hemolysis [58]. CD8 + cytotoxic T cells are thought to contribute to pathogenesis of IMHD, possibly through several mechanisms such as the direct cytotoxic effects and secretion of inflammatory cytokines including IL-17 [11]. Clonal expansion of CD8 + cells has been noted in some people with AIHA and ITP [12,13,59]. The dogs in the IMHD group were not age, sex, or breed matched to the control group. Proportions of lymphocyte subsets can vary with age and breed and might contribute to some of the observed differences between the populations [54,55].

The present study examined lymphocyte subsets in dogs diagnosed with IMHD during the first 2–4 days following the start of their treatment. These results were then compared to both the dogs’ lymphocyte concentration before treatment and to that found in healthy dogs. People with untreated chronic ITP showed alterations in T cell subsets compared to healthy individuals, including an increase in Th17 cells, a decrease in T regulatory cells, and a higher ratio of T helper 1 to T helper 2 cells. However, after 4 days of dexamethasone treatment, the T cell subsets in people with ITP resembled those of healthy individuals [60]. While it was interesting to see in the present study that the absolute number of CD4 + cells appeared to be increasing on Day 4 compared to earlier time points, this warrants further investigation with larger sample numbers to determine if it is a universal finding. Unfortunately, the low number of samples collected on Day 4 of the study precluded statistical analysis of data from this time point. Future study should also measure lymphocyte subsets at later time points to allow a comprehensive assessment of the effects of immunosuppressive therapy.

Commensurate with relatively few CD4+ and CD8 + lymphocytes was a relatively high proportion of CD4-/CD8- lymphocytes in dogs with IMHD. These double negative lymphocytes could be B lymphocytes or non-B/non-T lymphocytes such as NK cells. Aberrant B cells that produce excessive autoantibody have been identified in IMHD in people [61–63]. B lymphocytes were not enumerated in this study but should be included in future studies through the inclusion of B cell markers such as CD20.

The present study had additional limitations. The sample size was relatively small and could have underpowered the statistical analysis of some findings. The IMHD population was comprised of severely affected dogs, as it was recruited from a tertiary referral hospital and 4 dogs died or were euthanized within the first 4 days of diagnosis. As the study included clinical patients with IMHA and ITP, patients and treatments were somewhat heterogeneous. The inclusion of secondary immunosuppressive agents in some patients, in addition the use of non-leuko-reduced blood products, could have impacted the results. The immunopathogenesis of these two conditions is not necessarily identical and therefore grouping them together for analysis could confound results. Comparison of immune system alterations in dogs with IMHD to those in healthy and otherwise ill dogs could further characterize changes as specific to immune mediated disease pathogenesis. Concanavalin A was used in the cell preparation protocol, intended to be a lymphocyte stimulus to increase cytokine production, especially IL-17. There are usually low levels of IL-17 + produced by circulating lymphocytes, and stimulation with various protocols has been recommended prior to flow cytometric detection [21,64]. However, while concanavalin A will likely bind to lymphocytes at 4oC, the signaling pathways for lymphocyte activation may not be well executed. Cell stimulation with concanavalin A would have optimally been at 37oC [65]. Therefore, different results may have obtained if cells were stimulated at 37oC. This is likely a large contributor to the low number of positive cell events for IL-17 + lymphocyte subsets in the present study, and the significance of these findings should be verified in future studies. The anti-IL-17 antibody used in the present study was previously reported to detected canine IL-17 by flow cytometry and ELISA and correlated with IL-17 gene expression and time course assays [21,66,67]. While these findings do not entirely assure specificity for canine IL-17, they provide substantial evidence of specificity. Measurement of serum IL-17 would have been a valuable complement, however, a suitable IL-17A ELISA was no longer commercially available at the time of this study [28]. Future study would benefit from a larger population to allow for separate analysis of IMHA and ITP subpopulations, the use of age, sex, and breed matched controls, evaluation of IL-17 and other cytokine levels and mRNA expression in the serum. Furthermore, the use of cell cultures from affected dogs could allow for a more comprehensive assessment of lymphocyte subpopulations in future studies.

In conclusion, we identified a relative paucity of T regulatory cells and relative abundance of IL-17 + lymphocytes in dogs with IMHA and ITP at certain time points. While additional detailed analysis of immune alterations remains to be performed, these findings substantiate that changes in T cells are a feature of IMHD in dogs.

## Supporting information

S1 Table Details of individual dogs in the IMHD and healthy study populations, including diagnostic and treatment information for dogs with IMHD. FS, female spayed; IMHA, immune mediated hemolytic anemia; ITP, immune thrombocytopenia; MC, male castrated; NA, not available; POCUS, point-of-care ultrasound; PRBC, packed red blood cells; WBC, white blood cell.
(XLSX)
